# Prevalence of patent foramen ovale in cryptogenic transient ischaemic attack and non-disabling stroke at older ages: a population-based study, systematic review, and meta-analysis

**DOI:** 10.1016/S1474-4422(18)30167-4

**Published:** 2018-07

**Authors:** Sara Mazzucco, Linxin Li, Lucy Binney, Peter M Rothwell, Oxford Vascular Study Phenotyped Cohort

**Affiliations:** a Centre for Prevention of Stroke and Dementia, Nuffield Department of Clinical Neuroscience, University of Oxford, Oxford, UK

## Abstract

**Background:**

Percutaneous closure of patent foramen ovale (PFO) has been shown to be superior to medical treatment alone for prevention of recurrent stroke after cryptogenic transient ischaemic attack or non-disabling stroke in patients aged 60 years or younger. The justification for trials in older patients with transient ischaemic attack or stroke depends on whether PFO is shown to be associated with cryptogenic events at older ages, for which existing evidence is conflicting, and on the population burden of PFO-associated events. Therefore, we did a population-based screening study using contrast-enhanced transcranial Doppler (bubble-TCD) to detect probable PFO as indicated by a right-to-left shunt (RLS); we also did a systematic review and meta-analysis to compare our results with previous studies.

**Methods:**

In this population-based study, nested in the Oxford Vascular Study (OXVASC), we established the prevalence of any RLS, and of large RLS (>20 microbubbles), in consecutive patients attending a rapid-access transient ischaemic attack and stroke clinic, or at 1-month follow-up after stroke unit admission, with transient ischaemic attack or non-disabling ischaemic stroke, comparing cryptogenic events with those of known cause (according to Trial of Org 10172 in Acute Stroke Treatment [TOAST] criteria). We stratified participants by age, and extrapolated data to the UK population. We also did a systematic review of published studies of PFO prevalence (using transthoracic or transoesophageal echocardiography or bubble-TCD) according to stroke subtype, which included older patients and reported age-specific results, and determined by meta-analysis (including the OXVASC data) the pooled odds ratio (95% CI) of finding PFO of any size in cryptogenic events compared with events of known cause, stratified by screening modality (transthoracic or transoesophageal echocardiography or bubble-TCD). The study protocol is registered with PROSPERO, number CRD42018087074.

**Findings:**

Among 572 consecutive patients with transient ischaemic attack or non-disabling stroke between Sept 1, 2014, and Oct 9, 2017 (439 [77%] patients aged >60 years, mean age 70·0 years [SD 13·7]), bubble-TCD was feasible in 523 patients (91%) of whom 397 were aged older than 60 years. Compared with those with transient ischaemic attack or stroke of known cause, patients with cryptogenic events had a higher prevalence of RLS overall (odds ratio [OR] 1·93, 95% CI 1·32–2·82; p=0·001), and in those aged older than 60 years (2·06, 1·32–3·23; p=0·001). When we pooled the OXVASC data with that from two previous smaller studies of bubble-TCD in patients aged 50 years or older, we found an association between RLS and cryptogenic events (OR 2·35, 95% CI 1·42–3·90; p=0·0009; p_heterogeneity_=0·15), which was consistent with the equivalent estimate from transoesophageal echocardiography studies (2·20, 1·15–4·22; p=0·02; p_heterogeneity_=0·02). No data on large RLS in patients with TOAST-defined cryptogenic events compared with other events were available from previous studies, but we found no evidence that the association was diminished in such cases. Of 41 patients with large RLS and cryptogenic transient ischaemic attack or non-disabling stroke in our study, 25 (61%) were aged older than 60 years, which extrapolates to 5951 patients per year in the UK (data from mid-2016).

**Interpretation:**

Bubble-TCD was feasible in most older patients with transient ischaemic attack or non-disabling stroke, the association of RLS with cryptogenic events remained at older ages, and the population burden of PFO-associated events is substantial. Randomised trials of PFO closure at older ages are required and should be feasible.

**Funding:**

National Institute for Health Research, Oxford Biomedical Research Centre, Wellcome Trust, and Wolfson Foundation.

## Introduction

The prevalence of patent foramen ovale (PFO) is increased in cryptogenic transient ischaemic attack and stroke,^1^, ^2^, ^3^ and three recent trials^4^, ^5^, ^6^ showed better outcomes after percutaneous closure than after medical treatment alone. These trials included patients aged 60 years or younger, with mostly non-disabling events and PFO or an associated interatrial septal aneurysm (appendix).^4^, ^5^, ^6^ Clinical trials in older patients would be justified if an association between PFO and cryptogenic cerebrovascular events was shown at older ages, but existing evidence is conflicting,^2^, ^7^, ^8^ and the need for more data from older patients has been highlighted,^9^ along with a need for a better evidence base on the most appropriate screening strategy.

**Research in context**

**Evidence before this study**

We searched MEDLINE for articles published before Sept 1, 2017, with no language restrictions, reporting on age-specific prevalence of patent foramen ovale (PFO) in cryptogenic stroke compared with strokes of known cause and including older patients. We used the terms “stroke”, “CVA”, “cryptogenic stroke”, “undetermined stroke”, “stroke of undetermined aetiology”, “embolic stroke of undetermined source”, “patent foramen ovale”, “PFO”, “atrial septal abnormality”, “interatrial septal abnormality”, and “right-to-left shunt”. We also hand-searched international registries, reference lists of systematic reviews, and appropriate journals. We found that published evidence on the older population (age range 40 to ≥55 years) was exclusively hospital-based, focusing mainly on major stroke, and was mostly based on transoesophageal echocardiography, which is not the ideal screening method as up to a third of older stroke patients cannot undergo this procedure. Overall, evidence on the association between PFO and cryptogenic events in older patients was heterogeneous, especially when pooling smaller studies. No population-based data were found, and only two small studies of 210 and 203 patients used contrast-enhanced transcranial Doppler (bubble-TCD) as a screening method.

**Added value of this study**

This is the first population-based study investigating the association between PFO and cryptogenic events in a large number of patients, irrespective of age, but with clinical characteristics otherwise more similar to patients enrolled in trials on PFO closure (ie, mainly non-disabling events) than in previous stroke unit-based studies. Our study showed that bubble-TCD is possible in most older patients with transient ischaemic attack or non-disabling stroke, providing a feasible PFO screening strategy to reduce the need for transoesophageal echocardiography. We showed that PFO is significantly associated with cryptogenic events in older patients, consistent with previous evidence from transoesophageal echocardiography-based studies. Extrapolation from our results suggests that the burden of large PFO-related events at the population level is high, with as many as 8477 patients in the UK having large PFOs and cryptogenic transient ischaemic attack or non-disabling stroke per year, most of whom (61%, 5951 patients) are older than 60 years.

**Implications of all the available evidence**

In view of the results of published trials of PFO closure in younger patients showing an advantage of closure over medical therapy alone in preventing recurrent strokes and our results showing that right-to-left shunt was associated with cryptogenic events in older patients, age restrictions on access to diagnostic or therapeutic procedures in older patients with cryptogenic transient ischaemic attack or stroke should not prevent the necessary further research and randomised trials of PFO closure at older ages.

Transoesophageal echocardiography has been considered the gold standard for diagnosis of PFO,^10^, ^11^ and has been used in several recent trials.^4^, ^5^, ^6^ However, up to a third of older patients (>60 years) with stroke cannot undergo this procedure because of severity of stroke, dysphagia, excessive gag reflex, or refusal of consent.^7^, ^12^ Alternatively, contrast-enhanced transcranial Doppler (bubble-TCD) is a non-invasive, bedside, repeatable technique with high sensitivity and specificity for PFO detection in younger patients (≤60 years) and controls,^13^ through identification of right-to-left shunt (RLS). A small proportion of RLSs are due to non-PFO sources, usually pulmonary shunts, but the majority (around 95%) of TCD-detected RLSs are shown to be due to PFO on transoesophageal echocardiography.^13^, ^14^ An unsuitable temporal bone window limits the use of bubble-TCD in 10% of cases, but transoccipital insonation of the posterior circulation is a sensitive alternative to minimise screening failure and detect RLS.^15^

We previously reported an apparently low prevalence of markers of possible cardioembolic cause in patients with cryptogenic transient ischaemic attack and stroke at all ages,^16^ but we did not systematically screen for RLS. Therefore, we did a bubble-TCD-based screening study for RLS in patients with transient ischaemic attack or non-disabling stroke in a large population-based cohort, and extrapolated data to the UK population. We also did a systematic review of published studies reporting the prevalence of PFO in cryptogenic transient ischaemic attack or stroke compared with other causes stratified by age, and pooled our data in a meta-analysis.

## Methods

### Study design and participants

Our study was nested in the Oxford Vascular Study (OXVASC),^17^ an ongoing population-based study of the incidence and outcome of all acute vascular events in a population of 92 728 individuals, irrespective of age, registered with 100 primary care physicians in nine practices in Oxfordshire, UK. Multiple methods of ascertainment are used to enrol patients with transient ischaemic attack or stroke, as detailed elsewhere (appendix).^17^ These methods include a daily, rapid-access transient ischaemic attack and stroke clinic, to which participating physicians and the local emergency department refer individuals with suspected transient ischaemic attack or non-disabling stroke. As part of the OXVASC phenotyped cohort, consecutive eligible patients attending this clinic with an acute event, or at 1-month follow-up after an inpatient admission, were enrolled in PFO screening. Patients were eligible for PFO screening if they attended the transient ischaemic attack or stroke clinic or the 1-month follow-up clinic, had a diagnosis of transient ischaemic attack or stroke, and were able to undergo bubble-TCD.

The OXVASC study and TCD assessment were approved by the local ethics committee and written informed consent was obtained from all participants, or assent was obtained from relatives in the case of cognitive impairment or speech difficulty.

### Procedures

Patients were assessed by a neurologist or stroke physician and all presentations and investigations were reviewed by the senior study neurologist (PMR). Demographic data, atherosclerotic risk factors (ie, male sex, history of hypertension, diabetes, smoking, or hypercholesterolaemia), and history of coronary or peripheral vascular disease were recorded during face-to-face interviews and cross-referenced with primary care records.^16^ Patients routinely had 12-lead electrocardiography (ECG) and routine blood testing (ie, full blood count, clotting, C-reactive protein, erythrocyte sedimentation rate, liver function, renal function, thyroid function, electrolytes, and lipid profile) after the event. All patients had MRI brain and vascular imaging when not contraindicated (3T MRI with time-of-flight magnetic resonance angiography [MRA] of the intracranial vessels and a contrast-enhanced MRA of the large neck arteries), or brain CT with contrast-enhanced CT angiography, or Duplex ultrasound if MRI was contraindicated. Patients with cryptogenic transient ischaemic attack or stroke, or those younger than 55 years, also had thrombophilia screening, vasculitis screening, and genetic tests when clinically indicated. Clinical investigation was completed with 5-day ambulatory ECG recording (R test) and transthoracic echocardiography.^16^

Cause of ischaemic events was classified according to the Trial of Org 10172 in Acute Stroke Treatment (TOAST) criteria.^18^ We classified events as cryptogenic if the diagnostic investigation included at least brain imaging, ECG, transthoracic echocardiography, and complete vascular imaging, and no clear cause was found. Events of known cause included cardioembolic events, large artery disease, small vessel disease, events of other cause, or events of multiple causes. We did not consider PFO alone as a criterion for cardioembolic stroke.^16^

Contrast-enhanced TCD (bubble-TCD) sonography (Doppler Box; Compumedics DWL, Singen, Germany) was done according to the Consensus Conference of Venice^19^ by one of two experienced operators (SM and LL), who were masked to the patient's clinical presentation. Agitated saline with addition of 0·5 mL of the patient's blood and 0·5 mL of air was used as a contrast agent in all cases according to accepted guidelines (appendix).^19^, ^20^ A large RLS was defined as a shunt with 20 or more microbubbles recorded. Since Nov 15, 2015, if a temporal bone window was not suitable for monitoring, the basilar artery was monitored through a transoccipital approach.^15^ Designation of RLS status was made at the time of assessment, and recordings were archived.

### Search strategy and selection criteria of the systematic review, and data extraction

We did a systematic review and meta-analysis according to the Meta-analysis of Observational Studies in Epidemiology (MOOSE) criteria.^21^ We aimed to include case-control studies, cohort studies, and population-based studies that included older patients and reported on age-specific prevalence of PFO in cryptogenic stroke and strokes of known cause, irrespective of imaging modality (transthoracic echocardiography, transoesophageal echocardiography, or bubble-TCD). We excluded case reports, but did not limit the search to English language studies.

We searched MEDLINE using the terms “stroke”, “CVA”, “cryptogenic stroke”, “undetermined stroke”, “stroke of undetermined aetiology”, “embolic stroke of undetermined source”, “patent foramen ovale”, “PFO”, “atrial septal abnormality”, “interatrial septal abnormality”, and “right-to-left shunt” for articles published before Sept 1, 2017. We also hand-searched reference lists of all articles identified in the electronic search, the publications related to the component databases of the Risk of Paradoxical Embolism (RoPE) study,^22^ and any previous systematic reviews. We also contacted experts in the field, and all screened abstracts and selected papers were in English. Two researchers (SM and LL) independently did the search, and each identified eligible studies. Any discrepancy in relation to inclusion was resolved by majority decision by a third reviewer (PMR). Two researchers (SM and LL) independently extracted data from eligible papers.

For studies published more than once (ie, duplicates), we included only the report with the most informative and complete data. In the extracted data, PFO was defined according to the protocol of each individual study. In general, for studies using transthoracic echocardiography or transoesophageal echocardiography, the definition of PFO was based on demonstration of RLS by appearance of contrast microbubbles in the left atrium within three to five cardiac cycles after right atrium opacification.^2^, ^7^, ^12^ For studies using bubble-TCD, a definition based on the current consensus was used.^19^, ^20^ Extracted information included PFO screening modality (transthoracic echocardiography, transoesophageal echocardiography, or bubble-TCD), type of event (stroke or transient ischaemic attack), setting, case enrolment (consecutive *vs* non-consecutive), cryptogenic event out of all events ratio, stroke subtype classification (TOAST *vs* other), mean age of the population, age stratification, and excluded cases with reasons for exclusion, when provided.

### Statistical analysis

In the OXVASC cohort, baseline characteristics and prevalence of PFO of any size and of large size were compared between cryptogenic events and events of known cause for all patients and for those older than 60 years, using the χ^2^ test for categorical variables and *t* test for continuous variables. The numbers of large PFO in patients with cryptogenic events were reported by age and extrapolated to the UK population (data from mid-2016) based on age-specific rates.^23^

For each study in the systematic review, we established the odds ratio (OR) for PFO of any size in cryptogenic events compared with events of known cause, stratified by screening modality (transthoracic echocardiography, transoesophageal echocardiography, or bubble-TCD). We also compared the detection rate (%) of PFO using different screening modalities stratified by stroke cause (cryptogenic *vs* known cause) and by age (<70 *vs* ≥70 years).

We derived pooled ORs (95% CI) by meta-analysis, including the OXVASC data, using the Mantel-Haenszel-Peto method (random-effects), with χ^2^ tests to assess heterogeneity between studies.

Statistical analyses were done using Review Manager 5·3, Stata 15, and SPSS 22. The study protocol is registered with PROSPERO, number CRD42018087074.

### Role of the funding source

The funders of the study had no role in study design, data collection, data analysis, data interpretation, or writing of the report. All authors had full access to all the data in the study and PMR had final responsibility for the decision to submit for publication.

## Results

Among 572 consecutive patients with transient ischaemic attack or non-disabling ischaemic stroke between Sept 1, 2014, and Oct 9, 2017 (439 [77%] patients aged >60 years, mean age 70·0 years [SD 13·7]), bubble-TCD was feasible in 523 (91%) patients. Of the 49 (9%) patients who did not undergo bubble-TCD (appendix), two (4%) were deemed unsuitable for contrast (agitated saline) injection because of pregnancy, 24 (49%) could not tolerate testing (too frail, could not tolerate supine position, or too anxious); seven (14%; between Sept 1, 2014, and Nov 15, 2015, before the start of the transoccipital approach) did not have a suitable temporal bone window, and 16 (33%) had issues related to cannulation (refused or failed cannulation). One patient with a large RLS reported an episode of visual aura without headache, similar to his usual migraine aura, shortly after the injection of microbubbles,^24^ but no other complications were reported.

Of the 523 patients who underwent bubble-TCD, 397 (76%) were aged older than 60 years and 264 (50%) had cryptogenic events. Patients with cryptogenic events had fewer vascular risk factors, lower prevalence of comorbid atherosclerotic disease, and were more likely to have presented with a transient ischaemic attack than were patients with events of known cause (table 1).

**Table 1:** Baseline characteristics in patients with cryptogenic events versus events of known cause

		**Overall**			**Age >60 years**		
		Cryptogenic (n=264)	Known cause (n=259)	p value	Cryptogenic (n=190)	Known cause (n=207)	p value
Age		67·3 (7·3)	71·9 (8·1)	<0·0001	74·0 (6·9)	77·3 (8·3)	<0·0001
Male sex		132 (50%)	151 (58%)	0·06	84 (44%)	118 (57%)	0·01
Index event				0·0001			0·002
	Transient ischaemic attack	199 (75%)	154 (59%)	..	150 (79%)	134 (65%)	..
	Ischaemic stroke	65 (25%)	105 (41%)	..	40 (21%)	73 (35%)	..
Previous vascular event							
	Myocardial infarction	10 (4%)	29 (11%)	0·002	9 (5%)	27 (13%)	0·004
	Peripheral vascular disease	3 (1%)	21 (8%)	0·001	2 (1%)	20 (10%)	0·0002
	Transient ischaemic attack	16 (6%)	39 (15%)	0·001	14 (7%)	39 (19%)	0·001
	Stroke	21 (8%)	42 (16%)	0·005	18 (9%)	37 (18%)	0·02
Known vascular risk factors							
	Hypertension	139 (53%)	164 (63%)	0·01	120 (63%)	144 (70%)	0·18
	Diabetes	33 (13%)	44 (17%)	0·15	28 (15%)	32 (15%)	0·84
	Hyperlipidaemia	95 (36%)	102 (39%)	0·42	81 (43%)	89 (43%)	0·94
	Valvular heart disease	9 (3%)	25 (10%)	0·006	8 (4%)	23 (11%)	0·01
	Cardiac failure	3 (1%)	17 (7%)	0·005	3 (2%)	16 (8%)	0·004
	Venous thrombosis	8 (3%)	14 (5%)	0·18	6 (3%)	14 (7%)	0·10
	Atrial fibrillation*	1 (<1%)	111 (43%)	<0·0001	1 (1%)*	102 (49%)	<0·0001
	History of smoking	135 (51%)	148 (57%)	0·17	94 (49%)	113 (55%)	0·31
	Current smoker†	35 (13%)	41 (16%)	0·4	17 (9%)	16 (8%)†	0·67

Data are mean (SD) or n (%). Data are stratified by age.

* Including both history of atrial fibrillation and new atrial fibrillation detected after the index event. One patient with previous history of atrial fibrillation had successful ablation and was in sinus rhythm in repeated 5-day ambulatory cardiac monitoring.

† Data missing for one patient.

Overall, we found RLS in 157 (30%) of 523 patients, and large RLS in 68 (13%) patients. Compared with patients with transient ischaemic attack or stroke of known cause, cryptogenic events had a higher prevalence of RLS overall (OR 1·93, 95% CI 1·32–2·82; p=0·001; table 2). Results were consistent when stratified by type of presenting event (transient ischaemic attack: 1·90, 1·19–3·04; p=0·01; ischaemic stroke: 1·98, 1·00–3·90; p=0·05; ischaemic stroke or transient ischaemic attack with corresponding acute lesion on brain imaging: 2·18, 1·18–4·02; p=0·01). We found the same association when analysis was restricted to patients aged older than 60 years (2·06, 1·32–3·23; p=0·001; table 2); this association was independent of RLS size (large size: 2·10, 95% CI 1·27–3·46; small size: 1·77, 1·07–2·92; p_difference_=0·76; patients aged >60 years with large RLS: 2·67, 1·40–5·09; small RLS: 1·72, 0·99–2·97; p_difference_=0·78) and remained so when cryptogenic events were compared separately with events of cardioembolic, large vessel, or small vessel cause (appendix).

**Table 2:** Prevalence of RLS in patients with cryptogenic events compared with patients with events of known cause

	**Cryptogenic**	**Known cause**	**Odds ratio (95% CI)**	**p value**
**RLS of any size**				
Age ≤60 years	29/74 (39%)	16/52 (31%)	1·45 (0·68–3·07)	0·33
Age >60 years	68/190 (36%)	44/207 (21%)	2·06 (1·32–3·23)	0·001
Total	97/264 (37%)	60/259 (23%)	1·93 (1·32–2·82)	0·001
**Large RLS only**				
Age ≤60 years	16/74 (22%)	12/52 (23%)	0·92 (0·39–2·15)	0·85
Age >60 years	25/190 (13%)	15/207 (7%)	1·94 (0·99–3·80)	0·05
Total	41/264 (16%)	27/259 (10%)	1·58 (0·94–2·66)	0·08

Data are n/N (%) unless otherwise indicated. Data are stratified by age and size of the shunt. RLS=right-to-left shunt.

If extrapolated to the UK population (data from mid-2016), 41 patients with transient ischaemic attack and non-disabling cryptogenic stroke and a large RLS (25 [61%] of whom were older than 60 years) projected 8477 cases annually in the UK, 5951 (70·2%) of whom would be older than 60 years (appendix).

Of the 976 records identified in the systematic review (appendix), we selected 30 potentially relevant papers, of which eight met our criteria for inclusion in our meta-analysis (table 3),^7^, ^8^, ^12^, ^25^, ^26^, ^27^, ^28^, ^29^ reporting age-specific prevalence of PFO in cryptogenic stroke and strokes of known cause in a population including older patients.

**Table 3:** Studies included in the meta-analysis

	**Screening modality**	**Event (% stroke)**	**Setting**	**Consecutive cases**	**Cryptogenic events/total events**	**TOAST criteria**	**Mean age (SD), years**	**Age groups, years**	**Excluded cases**
**Hospital-based**									
Di Tullio et al (1992)^25^	Transthoracic echocardiography	Stroke	Neurology department	No	45/146 (31%)	No	61·8 (15·3)	<55 and ≥55	34% not referred for transthoracic echocardiography; 6·8% with inadequate transthoracic echocardiography
Hausmann et al (1992)^26^	Transoesophageal echocardiography	Transient ischaemic attack or stroke (59·2%)	Not reported	Not reported	65/103 (63%)	No	52 (10)	<40 and ≥40	Not reported
Jones et al (1994)^27^	Transoesophageal echocardiography	Transient ischaemic attack or stroke (90·5%)	Hospital admission	Yes	71/220 (33%)	No	66 (13)	<50 and 50–69	27·6%
Handke et al (2007)^7^	Transoesophageal echocardiography	Stroke	Stroke unit or intensive care unit	Yes	227/503 (45%)	Yes	62·2 (13·1)	<55 and ≥55	15·6%
De Castro et al (2010)^12^	Transoesophageal echocardiography	Transient ischaemic attack or stroke (62·1% major stroke)	Stroke unit	Yes	403/660 (61%)	Yes	64·4 (13·5)	<55 and ≥55	38·9%
Force et al (2008)^8^	Transoesophageal echocardiography	Transient ischaemic attack or stroke	Stroke unit	Not reported	62/132 (47%)	Not reported	70·7 (8·6)	≥55	Not reported
Yeung et al (1996)^28^	Bubble-TCD	Transient ischaemic attack or stroke (70·5%)	Hospital admission	Yes	116/210 (55%)	No	Men: 65 (range 12–86); Women: 63 (23–86)	≤50, >50, and ≥70	51%
Serena et al (1998)^29^	Bubble-TCD	Transient ischaemic attack or stroke (71·2%)	Neurology department	Yes	53/203 (26%)	No	64·8 (12·3)	<50, 51 to <70, and ≥70	20·9% (no temporal bone window) plus 1·9% (no Valsalva done)
**Population-based**									
OXVASC (2017)	Bubble-TCD	Transient ischaemic attack or non-disabling stroke (32·5%)	Stroke or transient ischaemic attack clinic service	Yes	264/523 (50%)	Yes	69·6 (13·4)	≤60, >60, <70, and ≥70	8·6%

TOAST=Trial of Org 10172 in Acute Stroke Treatment. Bubble-TCD=contrast-enhanced transcranial Doppler. OXVASC=Oxford Vascular Study.

**Figure 1:**
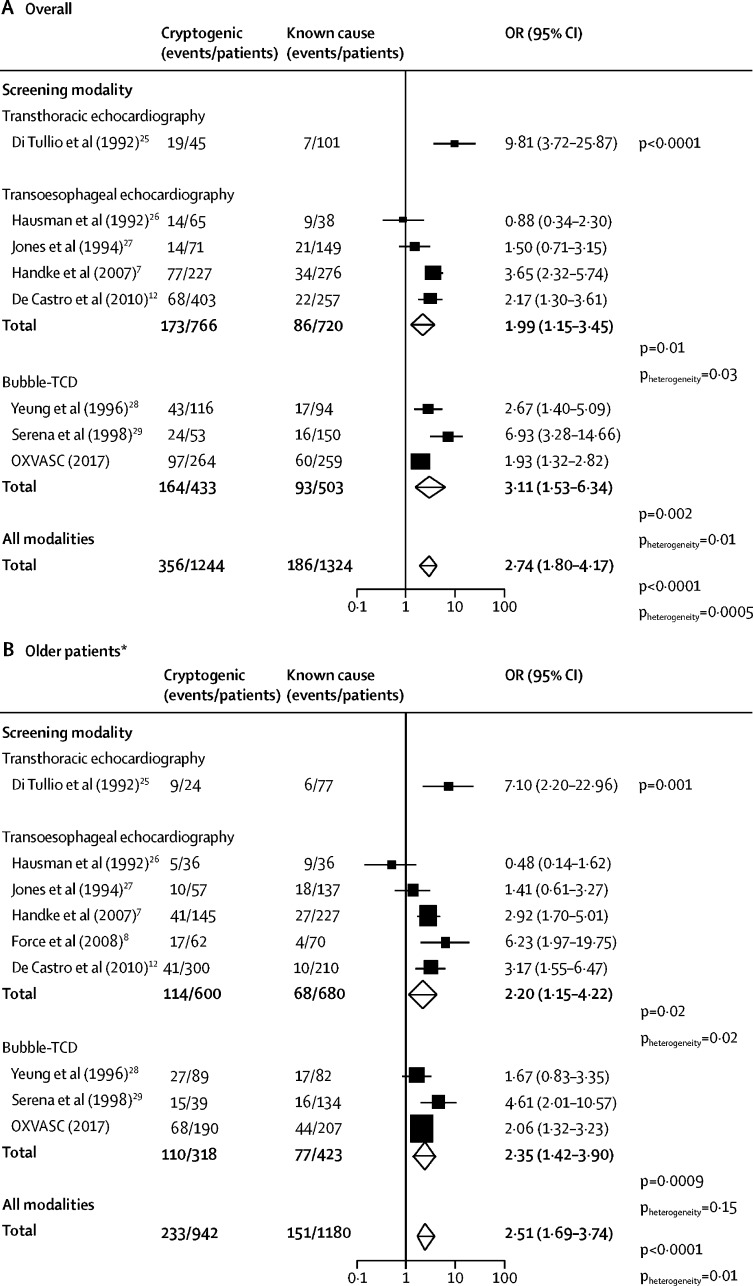
Prevalence of PFO in patients with cryptogenic events compared with patients with events of known cause Meta-analyses of the prevalence of PFO in patients with cryptogenic events compared with patients with events of known cause, stratified by imaging modalities, (A) overall and (B) in older patients, according to study author's definition. PFO=patent foramen ovale. OR=odds ratio. Bubble-TCD=contrast-enhanced transcranial Doppler. OXVASC=Oxford Vascular Study. *Age cutoff points for the older group in different studies ranged between 40 and 60 years.

Existing studies were exclusively hospital-based and predominantly based on transoesophageal echocardiography; overall, when pooling with results from the OXVASC study, an association between PFO and cryptogenic events was consistently shown for all screening modalities (figure 1). The association between RLS and cryptogenic events in patients older than 60 years found in the OXVASC study (based on 112 detected PFOs) was consistent with two previous smaller bubble-TCD studies of older age groups (based on 44^28^ and 31^29^ PFOs; figure 1), yielding a highly significant pooled estimate (OR 2·35, 95% CI 1·42–3·90; p=0·0009; p_heterogeneity_=0·15). This estimate was also consistent with that derived from studies of transoesophageal echocardiography (2·20, 1·15–4·22; p=0·02; figure 1), although the transoesophageal echocardiography estimate was heterogeneous (p_heterogeneity_=0·02).

**Figure 2:**
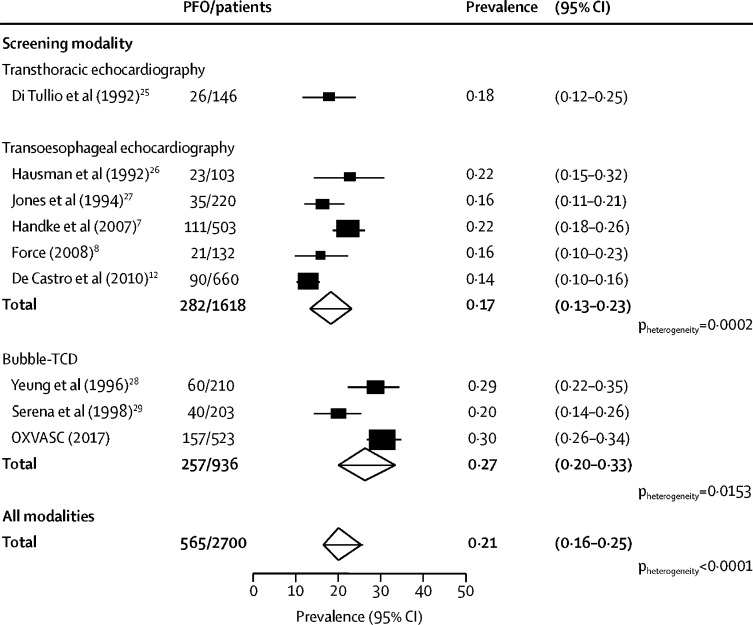
Meta-analyses of the prevalence of PFO stratified by screening modality PFO=patent foramen ovale. Bubble-TCD=contrast-enhanced transcranial Doppler. OXVASC=Oxford Vascular Study.

The prevalence of RLS suggested by bubble-TCD was higher than in studies based on transoesophageal echocardiography (0·27, 95% CI 0·20–0·33 *vs* 0·17, 0·13–0·23; p<0·0001; figure 2), both in patients with cryptogenic events (pooled prevalence 0·38, 0·33–0·43, for bubble-TCD studies *vs* 0·24, 0·16–0·32, for transoesophageal echocardiography studies; p<0·0001) and in strokes of known cause (0·17, 0·10–0·25, for bubble-TCD studies *vs* 0·11, 0·07–0·16, for transoesophageal echocardiography; p<0·0001; appendix). This difference in detected prevalence was also present in patients older than 70 years in OXVASC and other studies^8^, ^27^, ^29^ that reported data (appendix).

## Discussion

We showed that bubble-TCD was feasible in most patients in a consecutive series of relatively unselected older individuals with transient ischaemic attack or non-disabling stroke, and that there was a significant association between RLS and cryptogenic events in the older population. We found few published studies concerning the association between PFO and cryptogenic events at older ages, mostly based on transoesophageal echocardiography. Although the absolute prevalence varied from study to study, the association of PFO with cryptogenic transient ischaemic attack or stroke was reasonably consistent, particularly in bubble-TCD studies.^28^, ^29^ No previous study reported data for large PFOs in patients with TOAST-defined cryptogenic events compared with other events, but we confirmed the association for large RLS in patients aged older than 60 years, with more than one in ten patients with a cryptogenic event having a large RLS.

To our knowledge, no evidence has been reported from randomised trials that closure of PFO is effective in secondary prevention of stroke in patients older than 60 years. However, there is evidence that presence of a PFO is associated with increased risk of recurrent stroke in this age group.^9^ Our findings on the potential burden of transient ischaemic attack or stroke associated with a large RLS (ie, about 6000 patients aged >60 years with cryptogenic transient ischaemic attack and non-disabling stroke every year in the UK) show that large cohort studies with older patients are feasible, and that recruitment into subsequent randomised trials of PFO closure would probably be achievable. However, in this research it will be important that older patients with transient ischaemic attack or stroke are included, and that we avoid the age-related under-investigation and treatment that has been shown previously for other interventions in routine practice.^30^, ^31^, ^32^ Although trials might show that PFO closure is less effective and carries a higher risk of complications in older patients than in younger patients, concerns about the balance of risk and benefit at older ages have often proved unfounded in relation to other interventions.^33^, ^34^

Large cohort studies and trials in older patients with PFO will only be possible if patients undergo screening in routine clinical practice, although current clinical guidelines usually advocate PFO screening in younger patients with cryptogenic transient ischaemic attack or stroke,^35^ and routine screening in other populations is discouraged.^36^, ^37^ However, although bubble-TCD is not a substitute for transoesophageal echocardiography, which is still necessary to confirm the site of RLS and associated interatrial septal anatomical features, the latter procedure is invasive, often requires sedation, is costly, and has rare but serious complications.^38^ We have shown that bubble-TCD is a feasible screening method to identify the smaller subset of patients in whom transoesophageal echocardiography might be indicated. Not all patients were able to undergo screening, although this happened randomly and was not a proper source of selection. Bubble-TCD is much better tolerated than transoesophageal echocardiography and very few of our patients were unsuitable because of lack of a temporal bone window, particularly after we introduced the transoccipital approach in these cases.^15^ Moreover, the few patients in our study who did not undergo bubble-TCD would also not be suitable for transoesophageal echocardiography because of respiratory problems, frailty, or issues with cannulation. Overall, by using bubble-TCD as a screening tool, we showed that only 41 (16%) of 264 patients with cryptogenic transient ischaemic attack or stroke had a large RLS. Given the difference in cost of the procedures (National Health Service tariff of £506·30 for transoesophageal echocardiography with sedation *vs* £45·97 for bubble-TCD done by a technician), the cost of bubble-TCD prescreening followed by transoesophageal echocardiography in selected cases would be £32 784, compared with £133 663 for transoesophageal echocardiography screening in all 264 patients with cryptogenic events and RLS in our study. Transoesophageal echocardiography might sometimes identify other cardiac abnormalities in patients with crypotogenic transient ischaemic attack or stroke, but few health-care systems offer routine transoesophageal echocardiography in patients older than 60 years.

In our systematic review of all studies of PFO screening at older ages, we showed that studies using bubble-TCD reported RLS rates that were about 40–50% higher than studies using transoesophageal echocardiography, which contrasts with previous direct comparisons of the two techniques.^13^, ^14^ Although no studies in our review systematically screened patients using both techniques, and some differences in rates might be in part explained by patient selection, the higher RLS rate in the bubble-TCD studies might have other causes. First, transoesophageal echocardiography is less sensitive to latent shunts than is bubble-TCD because of the difficulty some older patients have doing a Valsalva manoeuvre under sedation. Second, the prevalence of non-cardiac RLS probably increases with age and so bubble-TCD could give a so-called false-positive for PFO in such cases; however, non-cardiac RLS might still be relevant to the cause of the stroke. We found a lower than expected prevalence of RLS in younger patients compared with some previous studies, which might reflect a lack of power due to the relatively small number of young patients enrolled, and our focus on transient ischaemic attack and non-disabling stroke. In the studies included in our analysis, there was heterogeneity between the age cutoff points for the younger and older group, ranging between 40 and 60 years of age (table 3). However, we found consistent results in analyses with cutoff points at more than 60 years and more than 70 years. We chose to classify the OXVASC study patients as older when aged over 60 years and younger when aged 60 years or less to be consistent with the recent trials, which included patients either younger than 60 years^5^ or aged 60 years and younger.^4^, ^6^

Our study has several strengths. First, we studied a consecutive series of unselected older patients. Second, we focused mainly on patients with non-disabling events, in line with trials in which PFO closure was shown to be beneficial.^4^, ^5^, ^6^ The other studies in our systematic review were based on admission to stroke units or intensive care units, and included many patients with major and disabling strokes.^7^, ^12^ However, our study does have some limitations. First, patients with RLS on bubble-TCD did not systematically undergo transoesophageal echocardiography, because of the absence of evidence on the benenfit of PFO closure at the time of our study. Second, for the same reason, we did not identify the presence of interatrial septal aneurysm. However, a policy of post-screening transoesophageal echocardiography in selected patients would identify interatrial septal aneurysm, although eligibility for closure of a large PFO was not dependent on the presence of interatrial septal aneurysm in any of the trials. Third, although we found large RLS to be associated with cryptogenic stroke at older ages, in the absence of further trials, it does not follow that routine closure would be justified. Moreover, not all trials of closure at younger ages showed benefit.^39^, ^40^ However, given that the diameter of PFO and the prevalence of venous thrombosis increase with age,^41^, ^42^ older patients might be more susceptible to paradoxical embolism associated with RLS, and some evidence suggests that the presence of PFO significantly increases the risk of recurrent ischaemic stroke or death in older patients with cryptogenic events than in younger patients with cryptogenic events.^9^

In conclusion, we found that bubble-TCD is feasible in most older patients with transient ischaemic attack or non-disabling stroke, with a higher rate of RLS than is usually reported in studies of transoesophageal echocardiography. We showed that large RLS is commonly associated with cryptogenic transient ischaemic attack or stroke and might be causal in some cases, such that cohort studies and trials of PFO closure in older patients are justified. Routine bubble-TCD is a feasible first-line screening modality for the detection of possible PFO and could facilitate further research.
