# Supplementary material for: Prevalence of patent foramen ovale in cryptogenic transient ischaemic attack and non-disabling stroke at older ages: a population-based study, systematic review, and meta-analysis
Source: Lancet Neurol. 2018 Jul;17(7):609–17. doi: 10.1016/S1474-4422(18)30167-4 (PMC6004554; doi:10.1016/S1474-4422(18)30167-4)

THE LANCET
Neurology

Supplementary appendix

This appendix formed part of the original submission and has been peer reviewed. We post it as supplied by the authors.

Supplement to: Mazzucco S, Li L, Binney L, Rothwell PM. Prevalence of patent foramen ovale in cryptogenic transient ischaemic attack and non-disabling stroke at older ages: a population-based study, systematic review, and meta-analysis. *Lancet Neurol* 2018; published online June 7. http://dx.doi.org/10.1016/S1474-4422(18)30198-4.

Prevalence of patent foramen ovale in cryptogenic TIA and non-disabling stroke at older ages: a population-based study, systematic review, and meta-analysis

Web extra material

Appendix 1 Inclusion criteria for randomised controlled trials comparing closure vs. medical treatment in patients with PFO and cryptogenic stroke published in 2017

Trial	Age	Definition of cryptogenic stroke	Inclusion criteria		Baseline characteristics	
			**mRS cut-off**	**PFO size**	**mRS distribution**	**PFO size**
REDUCE	18-59	Clinical TIA with imaging confirmed infarction (9.5%) or IS (90.5%)	≤2	All size	NR	81.3% moderate/large
CLOSE	16-60	Imaging confirmed ischaemic stroke regardless of the duration of symptoms	≤3	All size	0-1(81.6%)	92.8% large
RESPECT	18-60	Clinical TIA with imaging confirmed infarction or IS	≤3	All size	NR	48.8% "substantial"†

TIA=transient ischaemic attack; IS=ischaemic stroke; mRS=modified Rankin Scale; PFO=Patent Foramen Ovale; † shunt size of grade 3.

Appendix 2 OXVASC methodology

Study population

The Oxford Vascular Study (OXVASC) is a prospective, population-based cohort study of all incident acute vascular events in all territories (transient ischaemic attack, stroke, acute coronary and peripheral vascular events).[1,2]

During the period of the current substudy, the OXVASC study population consisted of all 92,728 individuals, irrespective of age, registered with 100 general practitioners (GPs) in nine general practices in Oxfordshire, UK. In the UK, general practices provide primary health care for registered individuals and hold a lifelong record of all medical consultations (from the National Health Service [NHS] and private health care), and details of treatments, blood pressure, and investigations. In Oxfordshire, an estimated 97% of the true residential population is registered with a general practice, with most non-registered individuals being young students. All participating practices held accurate age-sex patient registers, and allowed regular searches of their computerised diagnostic coding systems. The practices had all collaborated on a previous population-based study, for which they were originally selected to be representative of the urban and rural mix and the deprivation range of Oxfordshire as a whole.[3] Based on the index of multiple deprivation (IMD), the population was less deprived than the rest of England, but had a broad range of deprivation.

The OXVASC population is 94% white people, 3% Asian, 2% Chinese, and 1% Afro-Caribbean.[4] The proportion of whites is similar to that of the UK as a whole (88% white) and to many other western countries (Australia - 90%; France - 91%; Germany - 93.9%).

Case ascertainment

After a 3-month pilot study, the study started on April 1, 2002, and is ongoing. Ascertainment combined prospective daily searches for acute events (hot pursuit) and retrospective searches of hospital-care and primary-care administrative and diagnostic coding data (cold pursuit).

Hot pursuit was based on:

1. A daily (weekdays only), urgent open-access “TIA clinic” to which participating general practitioners (GPs) and the local accident and emergency department (A&E) send all individuals with suspected TIA or stroke whom they would not normally admit to hospital, with alternative on-call review provision at weekends. Patients too frail to attend are assessed at their residence by a study nurse or doctor.
2. Daily searches and case note review of admissions to the Emergency Assessment Unit, Medical Short Stay Unit, Coronary Care Unit and Cardiothoracic Critical Care Unit, Cardiology, Cardiothoracic, and Vascular Surgery wards, Acute Stroke Unit, Neurology ward and all other general wards when indicated.
3. Daily searches of the local A&E and eye hospital attendance registers.
4. Daily identification via the Bereavement Office of patients dead on arrival at hospital or who died soon after.
5. Daily searches of lists of all patients from the study population in whom a troponin-I level had been requested.
6. Daily assessment of all patients undergoing diagnostic coronary, carotid and peripheral angiography, angioplasty, stenting or vascular surgical procedures in any territory to identify both total burden of vascular invention and any potential missed prior acute events.

Cold pursuit procedures were:

1. Frequent visits to the study practices and monthly searches of practice diagnostic codes.
2. Monthly practice-specific list of all patients admitted to all acute and community NHS hospitals.
3. Monthly listings of all referrals for brain or carotid imaging studies performed in local hospitals.
4. Monthly reviews of all death certificates and coroners reports to review out-of-hospital deaths.
5. Practice-specific listings of all ICD-10 death codes from the local Department of Public Health.

Patients found on GP practice searches who have an event whilst temporarily out of Oxfordshire are included, but visitors who were not registered with one of the study practices are excluded. A study clinician assessed

patients as soon as possible after the event in the hospital or at home. Informed consent was sought, if possible, or assent was obtained from a relative.

Data are collected using event-specific forms, for TIA and stroke, acute coronary syndrome or acute peripheral vascular events. Standardised clinical history and cardiovascular examination are recorded. Information recorded from the patient, their hospital records and their general practice records includes details of the clinical event, medication, past medical history, all investigations relevant to their admission (including blood results, electrocardiography, brain imaging and vascular imaging-duplex ultrasonography, CT-angiography, MR-angiography or DSA) and all interventions occurring subsequent to the event.

If a patient died before assessment, we obtained an eyewitness account of the clinical event and reviewed any relevant records. If death occurred outside the hospital or before investigation, the autopsy result was reviewed. Clinical details are sought from primary care physicians or other clinicians on all deaths of possible vascular aetiology.

All surviving TIA and stroke patients are followed-up face-to-face at 1, 6, 12, 60 and 120 months after the initial event by a research nurse or physician and all recurrent vascular events were recorded together with the relevant clinical details and investigations. If face-to-face follow up is not possible, telephone follow-up is performed or enabled via the general practitioner. All recurrent vascular events that presented to medical attention would also be identified acutely by ongoing daily case ascertainment within OXVASC. If a recurrent vascular event was suspected at a follow-up visit or referred by the GPs to clinic or admitted, the patient was re-assessed and investigated by a study physician.

Definitions of events

Although new definitions for stroke and TIA have been suggested recently,[4,5] in order to enable comparison with previous studies, the classic definitions of TIA and stroke are used throughout.[6] A stroke is defined as rapidly developing clinical symptoms and/or signs of focal, and at time global (applied to patients in deep coma and to those with subarachnoid haemorrhage), loss of brain function, with symptoms lasting more than 24 hours or leading to death, with no apparent cause other than that of vascular origin.[6] A TIA is an acute loss of focal brain or monocular function with symptoms lasting less than 24 hours and which is thought to be caused by inadequate cerebral or ocular blood supply as a result of arterial thrombosis, low flow or embolism associated with arterial, cardiac or haematological disease.[4] All diagnoses were reviewed by a senior neurologist (PMR). With the high rate (97%) of imaging or autopsy in OXVASC, strokes of unknown type were coded as ischaemic.

References

1. Rothwell PM, Coull AJ, Giles MF, et al. Change in stroke incidence, mortality, case-fatality, severity, and risk factors in Oxfordshire, UK from 1981 to 2004 (Oxford Vascular Study). Lancet 2004; 363: 1925–33.
2. Rothwell PM, Coull AJ, Silver LE, Fairhead JF, Giles MF, Lovelock CE, Redgrave JNE, Bull LM, Welch SJV, Cuthbertson FC, Binney LE, Gutnikov SA, Anslow P, Banning AP, Mant D, Mehta Z for the Oxford Vascular Study. Population-based study of event-rate, incidence, case fatality and mortality for all acute vascular events in all arterial territories (Oxford Vascular Study). Lancet 2005; 366: 1773-83.
3. Bamford J, Sandercock P, Dennis M, Burn J, Warlow C. A prospective study of acute cerebrovascular disease in the community: the Oxfordshire Community Stroke Project--1981-86. 2. Incidence, case fatality rates and overall outcome at one year of cerebral infarction, primary intracerebral and subarachnoid haemorrhage. *J Neurol Neurosurg Psychiatry* 1990;53:16-22.
4. Easton JD, Saver JL, Albers GW, et al. Definition and evaluation of transient ischemic attack: a scientific statement for healthcare professionals from the American Heart Association/American Stroke Association Stroke Council; Council on Cardiovascular Surgery and Anesthesia; Council on Cardiovascular Radiology and Intervention; Council on Cardiovascular Nursing; and the Interdisciplinary Council on Peripheral Vascular Disease. The American Academy of Neurology affirms the value of this statement as an educational tool for neurologists. *Stroke* 2009;40:2276-2293.
5. Sacco RL, Kasner SE, Broderick JP, et al. An updated definition of stroke for the 21st century: a statement for healthcare professionals from the American Heart Association/American Stroke Association. *Stroke* 2013;44:2064-2089.
6. Hatano S. Experience from a multicentre stroke register: a preliminary report. *Bulletin of the World Health Organization* 1976;54:541-553.

Appendix 3 protocol for bubble-TCD performance and interpretation according to Venice Consensus conference

With the patient resting supine with the right arm (preferentially used for injection) in the horizontal position; using an 18-gauge needle into the right cubital vein, and a three-way stopcock connected to two 10-ml syringes, one containing 9 ml of 0.9% saline, and one containing 0.5 ml air aspirated through a bacterial filter and 0.5 ml of the patient's blood. Air and saline were energetically exchanged between the syringes at least ten times, and the obtained mixture was injected as a bolus. Injection was repeated twice, and the second time a Valsalva manoeuvre was performed 5 seconds after injection of the agitated saline, unless a relevant shunt was already detected with the first administration of the contrast agent.

Reference

1. Jauss M, Zanette E. Detection of right-to-left shunt with ultrasound contrast agent and transcranial Doppler sonography. Cerebrovasc Dis 2000; 10: 490–496.

2. Mojadidi MK, Zhang L, Chung Y, et al. Transcranial Doppler: Does Addition of Blood to Agitated Saline Affect Sensitivity for Detecting Cardiac Right-to-Left Shunt? *Echocardiography* 2016; **33**:1219-27.

Appendix 4 Baseline characteristics of patients that did not undergo TCD-bubble test

**Patients characteristics (N=49)**	**N(%)**
Age, mean /SD	75.5/16.7
Male sex	19 (38.8)
**Index event**	
Transient ischaemic attack	30 (61.2)
Ischaemic stroke	19 (38.8)
**Previous vascular events**	
Myocardial infarction	6 (12.2)
Peripheral vascular disease	2 (4.1)
Transient ischaemic attack	6 (12.2)
Stroke	10 (20.4)
**Known vascular risk factors**	
Hypertension	32 (65.3)
Diabetes	10 (20.4)
Hyperlipidaemia	13 (26.5)
Valvular disease	5 (10.2)
Cardiac failure	3 (6.1)
Atrial fibrillation*	9 (18.4)
History of smoking	20 (40.8)
Current smoker §	5 (10.2)

Data are presented as numbers (%) unless otherwise stated.
*Including both history of atrial fibrillation and new atrial fibrillation detected after the index event. One patient with previous history of atrial fibrillation had successful ablation and was in sinus rhythm in repeated 5-day ambulatory cardiac monitoring.
§Data missing for one patient.

Appendix 5 Associations of PFO and cryptogenic events vs. other individual stroke aetiology (TOAST classification) overall and in patients aged > 60 years

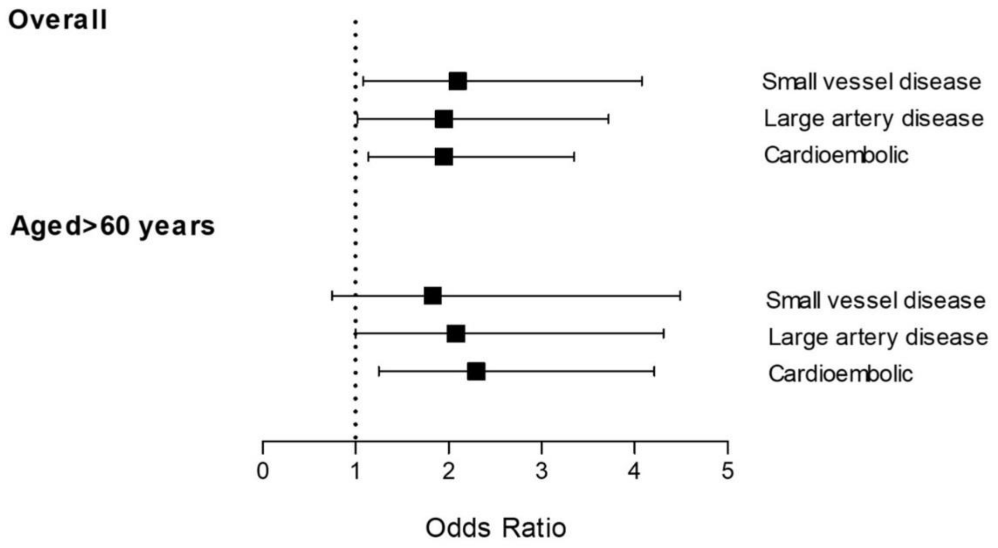

Each line represents the odds ratio of having PFO in cryptogenic events vs. individual stroke aetiology with 95%CI

Appendix 6 Age-specific numbers of large PFO in patients with cryptogenic TIA or non-disabling stroke standardised to the 2016 UK population

Age	Number/<br>number at risk	Rate<br>(95%CI)	UK population<br>(mid-2016)	Expected number (per year)
0-34	1/44547.3	0.01 (0,0.04)	28628400	184
35-44	3/12943.8	0.07 (0.01,0.19)	8353600	553
45-54	5/13176.5	0.11 (0.04,0.25)	9251100	1003
55-64	9/10845.5	0.24 (0.11,0.45)	7600900	1802
65-74	14/8794.8	0.45 (0.25,0.76)	6488600	2951
75-84	8/5394.3	0.42 (0.18,0.83)	3761200	1594
85+	1/2318.7	0.12 (0,0.69)	3170900	391
**Total**	**41/98020.9**	0.12 (0.09,0.16)	67254700	8477†

† Number rounded based on the sum of all expected number with 2 decimal places.

Appendix 7 Flow chart for systematic review

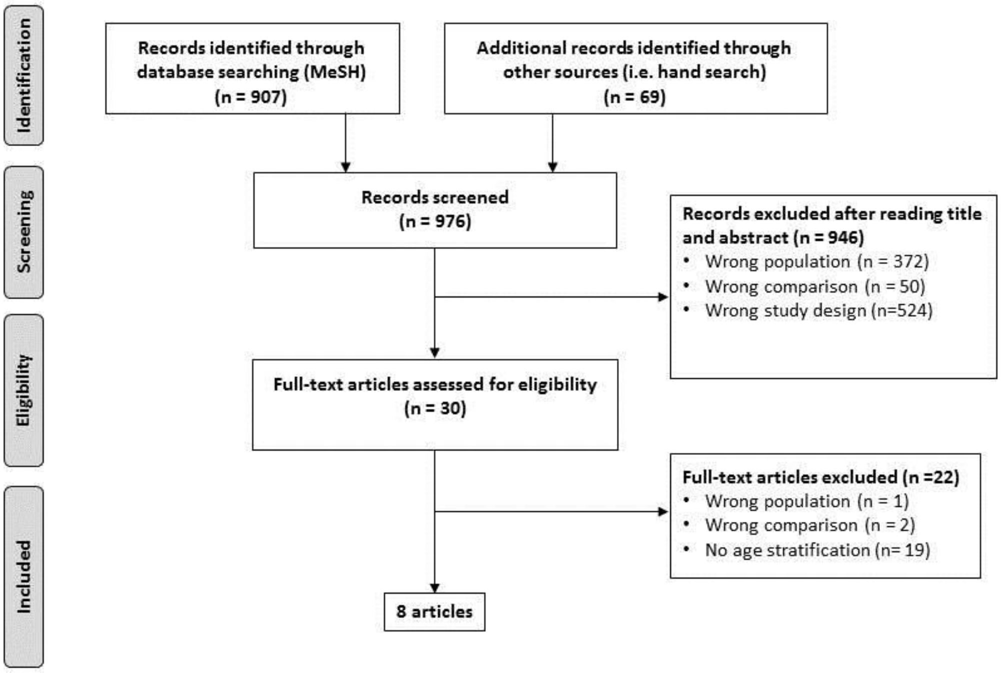

Appendix 8 Meta-analyses of the prevalence of PFO stratified by stroke aetiology and by screening modality.

Stroke subtypes	PFO / Patients	Prevalence	95% CI
**Cryptogenic stroke**			
**TTE**			
Di Tullio 1992	19 / 45	0.42	0.28-0.57
**TOE**			
Hausmann 1992	14 / 65	0.22	0.12-0.32
Jones 1994	14 / 71	0.20	0.10-0.29
Handke 2007	77 / 227	0.34	0.28-0.40
Force 2008	17 / 62	0.27	0.16-0.39
De Castro 2010	68 / 403	0.17	0.13-0.21
Total	190 / 828	0.24	0.16-0.32
**TCD**			
Yeung 1996	43 / 116	0.37	0.28-0.46
Serena 1998	24 / 53	0.45	0.32-0.59
OXVASC 2017	97 / 264	0.37	0.31-0.43
Total	164 / 433	0.38	0.33-0.43
**TOTAL**	**373 / 1306**	**0.31**	**0.23-0.38**
		Significance: p<0.0001	
		Heterogeneity: p<0.0001	
**Stroke of known aetiology**			
**TTE**			
Di Tullio 1992	7 / 101	0.07	0.02-0.12
**TOE**			
Hausmann 1992	9 / 38	0.24	0.10-0.37
Jones 1994	21 / 149	0.14	0.09-0.20
Handke 2007	34 / 276	0.12	0.08-0.16
Force 2008	4 / 70	0.06	0.00-0.11
De Castro 2010	16 / 257	0.06	0.03-0.09
Total	84 / 790	0.11	0.07-0.16
**TCD**			
Yeung 1996	17 / 97	0.18	0.10-0.25
Serena 1998	16 / 150	0.11	0.06-0.16
OXVASC 2017	60 / 259	0.23	0.18-0.29
Total	93 / 506	0.17	0.10-0.25
**TOTAL**	**184 / 1397**	**0.13**	**0.09-0.17**
		Significance: p<0.0001	
		Heterogeneity: p<0.0001	

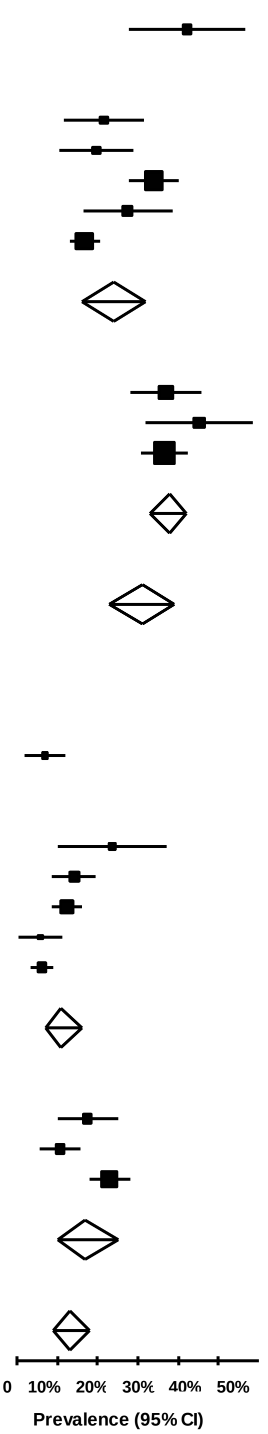

TTE=transthoracic echocardiography, TOE=transoesophageal echocardiography, TCD=transcranial Doppler, PFO=patent foramen ovale

Appendix 9 Prevalence of PFO stratified by stroke aetiology, imaging modalities and patient age

TOE=transoesophageal echocardiography, TCD=transcranial Doppler, PFO=patent foramen ovale

	Events / Patients	Prevalence	95% CI
**Cryptogenic stroke**			
**TOE**			
**<70 years**			
Jones 1994	9 / 44	0.20	0.09-0.32
**≥ 70 years**			
Jones 1994	5 / 27	0.19	0.04-0.33
Force 2008	5 / 16	0.31	0.09-0.54
Total	10 / 43	0.24	0.12-0.37
**TCD**			
**<70 years**			
Serena 1998	16 / 35	0.46	0.29-0.62
OXVASC 2017	52 / 127	0.41	0.32-0.50
Total	68 / 162	0.42	0.35-0.50
**≥ 70 years**			
Serena 1998	8 / 18	0.44	0.22-0.68
OXVASC 2017	45 / 137	0.33	0.25-0.41
Total	53 / 155	0.34	0.27-0.42
**Stroke of known aetiology**			
**TOE**			
**<70 years**			
Jones 1994	13 / 71	0.18	0.09-0.27
**≥ 70 years**			
Jones 1994	8 / 78	0.10	0.04-0.17
Force 2008	1 / 34	0.03	0.00-0.12
Total	9 / 112	0.08	0.02-0.15
**TCD**			
**<70 years**			
Serena 1998	8 / 88	0.09	0.04-0.16
OXVASC 2017	26 / 95	0.27	0.19-0.37
Total	34 / 183	0.16	0.02-0.38
**≥70 years**			
Serena 1998	8 / 62	0.13	0.05-0.21
OXVASC 2017	34 / 164	0.21	0.15-0.27
Total	42 / 226	0.18	0.11-0.25

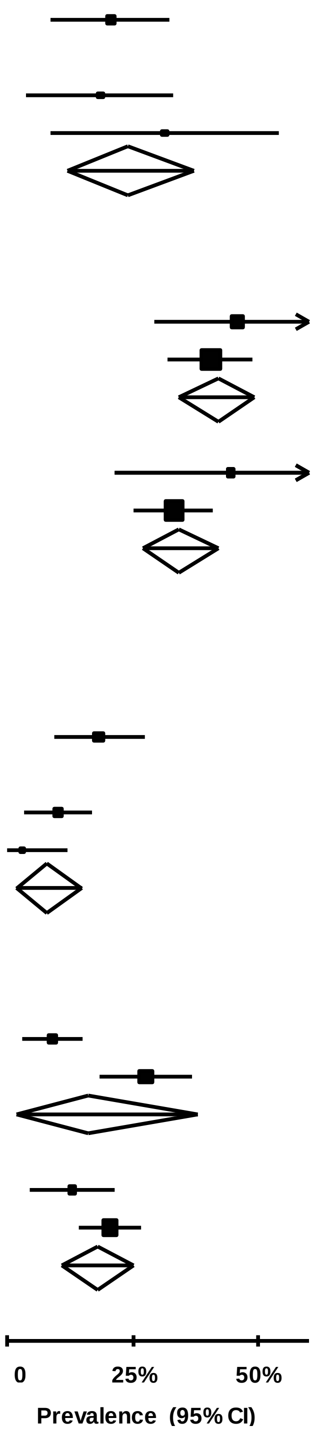

Supplement: Supplementary appendix [file mmc1.pdf]
